# Hospitalisation after birth of infants: cross sectional analysis of potentially avoidable admissions across England using hospital episode statistics

**DOI:** 10.1186/s12887-018-1360-z

**Published:** 2018-12-20

**Authors:** Eleanor Jones, Beck Taylor, Gavin Rudge, Christine MacArthur, Deepthi Jyothish, Doug Simkiss, Carole Cummins

**Affiliations:** 1 Institute of Applied Health Research, University of Birmingham, Edgbaston, Birmingham, B15 2TH England; 2 Birmingham Women’s and Children’s NHS Foundation Trust, Steelhouse Lane, Birmingham, B4 6NH England; 3 Division of Mental Health and Wellbeing, Warwick Medical School, University of Warwick, Coventry, UK

**Keywords:** Infant admission, Avoidable readmission, Postnatal care

## Abstract

**Background:**

Admissions of infants in England have increased substantially but there is little evidence whether this is across the first year or predominately in neonates; and for all or for specific causes. We aimed to characterise this increase, especially those admissions that may be avoidable in the context of postnatal care provision.

**Methods:**

A cross sectional analysis of 1,387,677 infants up to age one admitted to English hospitals between April 2008 and April 2014 using Hospital Episode Statistics and live birth denominators for England from Office for National Statistics. Potentially avoidable conditions were defined through a staged process with a panel.

**Results:**

The rate of hospital admission in the first year of life for physiological jaundice, feeding difficulties and gastroenteritis, the three conditions identified as potentially preventable in the context of postnatal care provision, increased by 39% (39.55 to 55.33 per 1000 live births) relative to an overall increase of 6% (334.97 to 354.55 per 1000 live births). Over the first year the biggest increase in admissions occurred in the first 0–6 days (RR 1.26, 95% CI 1.24 to 1.29) and 85% of the increase (12.36 to 18.23 per 1000 live births) in this period was for the three potentially preventable conditions.

**Conclusions:**

Most of the increase in infant hospital admissions was in the early neonatal period, the great majority being accounted for by three potentially avoidable conditions especially jaundice and feeding difficulties. This may indicate missed opportunities within the postnatal care pathway and given the enormous NHS cost and parental distress from hospital admission of infants, requires urgent attention.

**Electronic supplementary material:**

The online version of this article (10.1186/s12887-018-1360-z) contains supplementary material, which is available to authorized users.

## Background

Hospital admissions, especially emergency ones place a huge cost on health services [1, 2] and there is evidence from studies using Hospital Episode Statistics (HES) data for England that emergency admissions of children have increased substantially. In children under 15 between 1999 and 2010 in England all emergency admissions increased with the greatest increase in infants: in 2010 over a third of infants had an admission some time in their first year [3]. While emergency admissions between 2006 and 2016 increased in all age categories 0–24, this was greatest in those under one [4]. Short-stay (< 2 days) unplanned admissions among children up to age 10 increased between 1996 to 2006, again with the greatest increase in children less than one [4]. A study of infant admission in England using HES data showed that between 2005 and 2014, 5.2% of infants were readmitted unexpectedly within 30 days of postnatal discharge and that the risk of readmission increased by 4.4% annually from 4.4% in 2005 to 6.3% in 2014 [5]. Whilst similar trends have been observed in Scotland [6] in the United States and Canada the proportion of hospital stays for children has decreased or remained relatively unchanged over the period 2000–2012 [5, 7].

Over the last 30 years, the postnatal length of stay in hospital in the UK has reduced considerably: in 1989–90, only 44% women were discharged within two days of giving birth compared to 81% women in 2016–17 [8]. Over the last decade, the number of women going home on the same calendar day that they gave birth has increased considerably from (16.5% in 2005/06 to 19.8% in 2016/17 [8]. Following discharge from hospital, women and babies also have fewer visits from community midwifery services before being discharged to the care of the community health visitor and GP [9, 10].

We wanted to know whether the changes to postnatal care provision coincided with the increase in infant admissions which in some cases may have been potentially avoided. We sought to investigate whether the increase in infant admissions was predominantly in the early neonatal period and whether it was confined to a sub-group of conditions more sensitive to the quantity and quality of postnatal care, and therefore amenable to intervention earlier in the care pathway. If findings showed this to be the case, the current five year national maternity review programme in England [11] would provide an opportunity to consider the potential for intervention.

## Methods

Data on all admissions to hospital in the first year of life across England from 1st April 2008 to 31st March 2014 from Hospital Episode Statistics (HES) were included. We developed clinical definitions of potentially avoidable conditions. Admission rates were calculated with denominator data on all live births from Office for National Statistics (ONS). Main outcomes were admissions to hospitals for potentially preventable conditions across different ages within the first year and overall admissions.

An anonymised extract of inpatient data from Hospital Episode Statistics (HES) for all NHS hospitals in England from 1st April 2008 to 31st March 2014 was obtained. HES collects routine demographic data, administrative information and clinical information based on World Health Organisation (WHO) ICD 10 (2008, 2010 and 2014 versions) and OPCS4 and is suitable for research purposes [12]. All admissions (planned and unplanned) of infants less than one year old at the start of their admission episode were extracted. Since the vast majority of infant admissions are unplanned, that is, emergencies, it was decided to include all admissions in these analyses. An inpatient admission was defined as a ‘continuous inpatient spell’ which is the continuous time spent in hospital from admission to discharge regardless of any within-hospital transfers [12]. This may have included several ‘episodes of care’ under different medical teams at various NHS care providers. Clinical diagnosis data were obtained from the final discharge episode of the spell. This method was chosen because using the diagnosis from the admission episode might underestimate the case-mix severity in multi-episode spells. The majority of inpatient spells only have one episode which is both the admission and discharge episode. Duplicate cases and cases with an implausible admission/discharge date were removed and readmissions were explored using the HES identification variable.

To avoid capturing routine admissions to the postnatal ward which frequently occur with a hospital birth, based on the HES data dictionary [12] cases with method of admission codes of ‘31 (admitted antenatally)’, ‘32 (admitted postnatally)’, i.e. immediately following delivery, ‘82 (the birth of a baby in this healthcare provider)’; or ‘83 (baby born outside the healthcare provider except when born at home as intended)’ were excluded. Also excluded were cases with episode type given as ‘Birth episode’; diagnosis ICD10 coded as ‘Z37-Z38’ (Singleton, born in Hospital) or admission source coded as ‘79’ (Babies born in or on the way to hospital) (Additional file 1).

The data recorded in HES for each admission included a code for infant age category on admission. Codes for gender, region of admission and ethnicity were also included and a score for social deprivation was assigned, allowing exploration of rate variations by these characteristics. Ethnicity within HES is self-reported and the 16 Census ethnic groups [13] were merged into 5 groups to avoid risk of de-anonymisation for any very small groups when merged with the ONS data: White (British, Irish, Any other white background), Asian (Indian, Pakistani, Bangladeshi, Any other Asian Background), Black (Caribbean, African, Any other black background), Other (White and Black Caribbean, White and Black African, White and Asian, Any other mixed Background, Chinese, Any other ethnic group), Not stated (not stated, missing/null).

Each infant admitted was assigned a Local Authority District and Government Office Region (GOR) of residence based on their lower super output area (LSOA) of residence. A LSOA is a small unit of United Kingdom census geography [14] and contains a mean resident population of approximately 1600 individuals [14]. An index of multiple deprivation 2010 score was assigned to each individual based on the LSOA [15]. The index of multiple deprivation (IMD) is an area based score that combines housing, social and economic indicators to indicate the level of deprivation in each area. The income domain score is the one that most accurately reflects material deprivation as it is based on the Government definition of poverty. These were converted into quintiles by subdividing the ranks of the 32,480 areas in England, quintile 1 being most deprived and quintile 5 least deprived.

Denominator data on all live births across England was provided by Office for National Statistics (ONS), providing frequencies of live births by financial year of birth, Region (mothers’ area of usual residence), gender, ethnicity (White, Black, Asian, Other, Not stated) and IMD quintile.

**Fig. 1:**
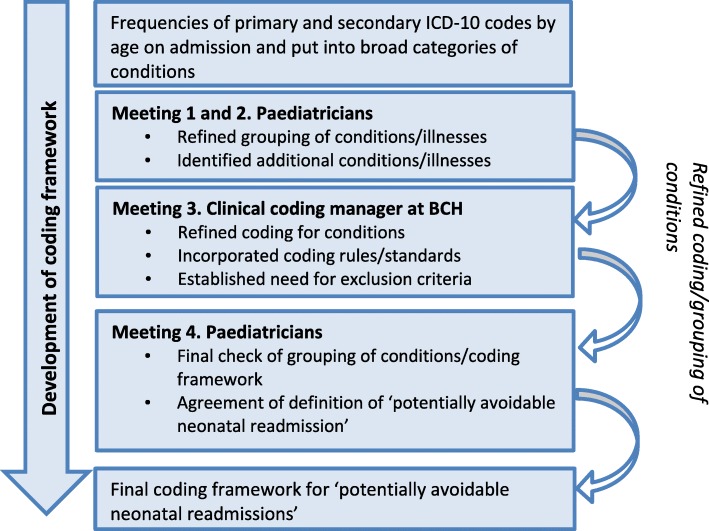
Process for identifying potentially avoidable admissions and development of the coding framework

Pre-specified definitions of ICD-10 codes of potentially avoidable admissions were produced before analysis of admission rates. The definition of potentially avoidable in this context was a condition or illness which could have been identified before postnatal discharge from hospital or in the community and adequately treated during birth hospitalisation or through community care services. The process for identifying conditions/illness that could be considered potentially avoidable within the HES dataset was undertaken with an advisory panel comprising a consultant general paediatrician, a consultant community paediatrician/professor of child health and a clinical coding manager at a specialist children’s hospital. The selection of conditions/illnesses and corresponding coding framework was developed in a four stage approach (Fig. 1). Firstly, frequencies of common illnesses/conditions with the relevant ICD-10 codes by age on admission were produced from the dataset and the paediatricians considered the clinical care pathways, in addition to the physiological and aetiological factors associated with the conditions. Secondly, the list of potentially avoidable conditions and corresponding coding framework was refined. Thirdly, discussion with an expert clinical paediatric coder identified specific HES diagnosis coding rules and standards relevant to the dataset. Finally, a formal list of conditions and corresponding ICD-10 codes was agreed (Additional file 2). Conditions identified as potentially avoidable were physiological jaundice, feeding difficulties and gastroenteritis and these were pre-specified as the main outcomes prior to data analysis. Potentially avoidable implies that although the infant may require admission to hospital at the point of contact with secondary care services, the risk of developing the illness or the severity of the illness may have been reduced had the problem been identified and an intervention taken place earlier.

Patient involvement was via the National Institute for Health Research Collaboration for Leadership in Applied Health Research and Care West Midlands Patient and Public Involvement Supervisory Committee.

### Analyses

SPSS (V.22) was used to analyse all infant admissions. Summary statistics were used to describe the proportion of avoidable infant admissions in 0–6 days and 7–28 days, 1 to under 3 months, 3 to under 6 months, 6 to under 9 months and 9 to 12 months after birth by condition/illness, ethnicity, deprivation indices, region in England and year of admission. Frequency of admissions by hospital trust was also explored in addition to exploring readmission rates. Unadjusted annual infant admission rates and annual rates for specific conditions and 95% confidence intervals were calculated by (N admissions for each year/N live births 2008–09) × 100. Change in admission rates were calculated as follows: (rate in 2013–14/rate in 2008–09) × 100. Where appropriate, Cochrane Armitage tests for trend were conducted to assess significance of the year on year trend over the 6 year period. A sample size calculation was not necessary due to the exploratory and descriptive nature of the study. The following sensitivity analyses were conducted: comparison of the rates of admissions by episodes of care versus spells of care and selecting the primary diagnosis code versus all diagnostic codes.

## Results

**Fig. 2:**
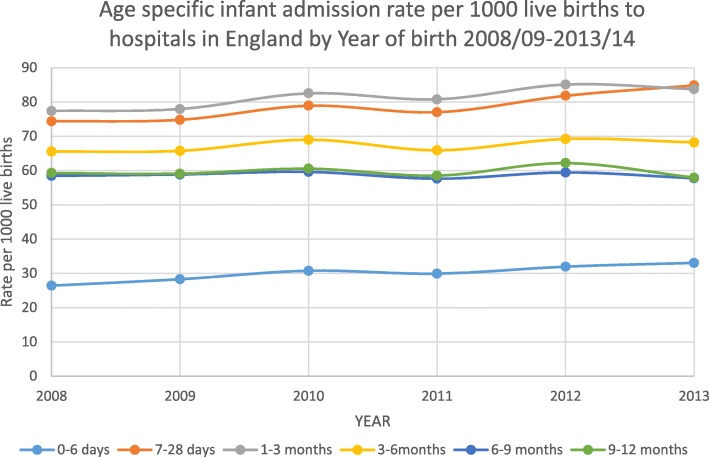
Age specific infant hospital admission rates in England per 1000 live births by year of birth 2008/09 to 2013/14

There were 1,387,677 admissions in the first year of life and 4,063,050 live births from 1st April 2008 to 31st March 2014. The overall rate of admission increased significantly over the period from 335·0 (95% CI 333·8–336·1) to 354·6 (95% CI 353·6–355·9) per 1000 live births (Table 1). Infants born in 2013/14 had 1·06 times the risk of being admitted to hospital within the first year of life compared to infants born in 2008/09 (Relative risk 1·06, 95% CI 1·05–1·06). Infants who had one admission were 47% more likely to be readmitted at least once more within the first year of life. The increase in admissions was most marked for the 0–6 day age category where admission rate increased from 26·39 per 1000 live births (95% CI 26·01–26·78) in 2008/09 to 33·31 per 1000 live births in 2013/14 (95% CI 32·88–33·74) (*P* < 0.0001). Infants born in 2013/14 had 1·26 times the risk of being admitted within the first 6 days of life compared with infants born in 2008/09 (Relative risk 1·26, 95% CI 1·24–1·29) (Fig. 2).

**Table 1:** Number and incidence (per 1000 live births) of infants admitted by year of birth and age group in England 2008/09–2013/14

YEAR	Total No. admissions	No. Live births	Rate* (95% CI)	Age specific rates of admissions per 1000 population (95% CI)					
				0–6 days	7–28 days	1–3 months	3–6 months	6–9 months	9–12 months
2008/09	223,735	667,932	334·97 (333·83–336·10)	26·39 (26·01–26·78)	47·96 (47·44–48·47)	77·38 (76·74–78·02)	65·56 (64·97–66·15)	58·43 (57·86–58·99)	59·25 (58·69–59·82)
2009/10	225,130	674,949	333·55 (332·43–334·68)	27·96 (27·56–28·35)	46·81 (46·30–47·31)	77·11 (76·47–77·74)	65·04 (64·45–65·63)	58·20 (57·65–58·76)	58·44 (57·88–59·00)
2010/11	235,288	682,892	344·55 (343·42–345·67)	30·07 (29·66–30·47)	48·82 (48·31–49·33)	80·69 (80·05–81·34)	67·49 (66·89–68·08)	58·27 (57·72–58·83)	59·20 (58·64–59·76)
2011/12	228,534	689,582	331·41 (330·30–332·52)	28·95 (28·55–29·34)	48·03 (47·52–48·53)	78·20 (77·56–78·83)	63·81 (63·24–64·39)	55·78 (55·24–56·32)	56·64 (56·09–57·18)
2012/13	240,090	685,174	350·41 (349·28–351·54)	31·14 (30·73–31·55)	50·66 (50·14–51·18)	82·94 (82·29–83·59)	67·46 (66·86–68·05)	57·91 (57·36–58·47)	60·59 (60·03–61·16)
2013/14	234,900	662,521	354·55 (353·59–355·89)	33·31 (32·88–33·74)	51·49 (50·96–52·02)	84·40 (83·73–85·07)	68·77 (68·16–69·38)	58·19 (57·63–58·76)	58·39 (57·82–58·93)
Cochran Armitage test for trend			775·7362 (*P* < 0·0001)	611·4452 (*P* < 0·0001)	166·0960 (*P* < 0·0001)	959·3922 (*P* < 0·0001)	58·8150 (*P* < 0·0001)	3·7260 (*P* = 0·0536)	0·0190 (*P* = 0·8904)

*per 1000 live births

Admission rates also varied considerably by ethnicity where the highest rate of admission was in the ‘not stated’ ethnicity category (528·22 per 1000 live births (95% CI 525·93–530·52) compared to 216·85 per 1000 live births (95% CI 215·11–218·60) in the Black ethnicity category.

The rate of admission for the *potentially avoidable conditions* increased by 39% from 39·79 to 55·33 per 1000 live births (Table 2). In the 0–6 day age category the increase in admissions to hospital for these three conditions from 12·36 to 18·23 per 1000 live births contributed 85% of the increase in admission rate. The rate of admission for infants under 7 days increased by 6·92 per 1000 live births (RR 1·26, 95% CI 1·24–1·29) however, once the potentially avoidable admissions were removed the rate only increased by 1·05 per 1000 live births (RR 1·07 95% CI 1·04–1·10) (Table 2).

**Table 2:** Frequency and rate (per 1000 live births) of admission for infants aged 0–6 days (overall and potentially preventable conditions (physiological jaundice, feeding difficulties and gastroenteritis))

YEAR	overall No. admissions	No. avoidable conditions	No. live births	rate^a^ overall admission	admission rate^a^ for potentially avoidable conditions
2008/09	17,629	8257	667,932	26·39	12·36
2009/10	18,869	8798	674,949	27·96	13·04
2010/11	20,534	9932	682,892	30·07	14·54
2011/12	19,962	10,313	689,582	28·95	14·96
2012/13	21,334	11,373	685,174	31·14	16·60
2013/14	22,067	12,079	662,521	33·31	18·23

^a^per 1000 live births

For *physiological jaundice* there were a total of 73,403 admissions over the study period, the rate of admission increasing from 16·30 (95% CI 16·00–16·61) to 22·35 (95% CI 21·99–22·70) admissions per 1000 live births (*P* < 0.0001) (Table 3). The admission rate in 2013/14 was 1·37 times the risk of being admitted in 2008/09 (RR 1·37 95% CI 1·34–1·.40), an absolute risk increase of 6 per 1000 live births. The increase was concentrated in the 0–6 day category where the admission rate rose from 8.40 to 12·45 per 1000 with statistically significant increases confined to the first 28 days. (Table 3) The duration of hospital admission for physiological jaundice was short with a median length of stay of 1·6 days. The vast majority of infants (94%) admitted for physiological jaundice had a hospital duration of ≤3 days.

**Table 3:** Number and incidence (per 1000 live births) of infant admissions for potentially preventable conditions for infants by Year and Age group on admission 2008/09–2013/14

YEAR	No. admissions	Rate ^a^ (95% CI)	Age specific rates of admissions per 1000 live births (95% CI)					
			0–6 days	7–28 days	1–3 months	3–6 months	6–9 months	9–12 months
Gastroenteritis								
2008/09	8108	12·14 (11·88–12·40)	0·02 (0·01–0·03)	0·38 (0·33–0·43)	1·77 (1·67–1·87)	2·48 (2·36–2·60)	3·37 (3·23–3·51)	4·11 (3·96–4·26)
2009/10	8257	12·23 (11·97–12·50)	0·01 (0·00–0·02)	0·35 (0·31–0·40)	1·80 (1·70–1·90)	2·43 (2·31–2·54)	3·53 (3·39–3·68)	4·11 (3·95–4·26)
2010/11	7785	11·40 (11·15–11·65)	0·01 (00·00–0·010	0·28 (0·24–0·32)	1·56 (1·46–1·65)	2·46 (2·34–2·58)	3·20 (3·07–3·34)	3·89 (3·75–4·04)
2011/12	7859	11·40 (11·15–11·65)	0·01 (0·00–0·01)	0·26 (0·22–0·30)	1·55 (1·46–1·64)	2·34 (2·23–2·45)	3·27 (3·14–3·41)	3·96 (3·81–4·11)
2012/13	15,451	22·55 (22·20–22·90)	0·04 (0·03–0·06)	0·58 (0·53–0·64)	3·61 (3·47–3·76)	4·58 (4·42–4·74)	6·24 (6·06–6·43)	7·49 (7·29–7·69)
2013/14	13,155	19·86 (19·52–20·19)	0·03 (0·02–0·05)	0·56 (0·50–0·61)	3·61 (3·47–3·75)	4·32 (4·16–4·47)	5·30 (5·12–5·47)	6·04 (5·86–6·23)
Cochran Armitage test for trend:		3170·9178 (*P* < 0·0001)	10·2750 (*P* = 0·013)	57·4530 (*P* < 0·0001)	805·7172 (*p* < 0·0001)	748·2772 (*P* < 0·0001)	742·1982 (*P* < 0·0001)	779·7762 (*P* < 0·0001)
Physiological jaundice								
2008/09	10,890	16·30 (16·00–16·61)	8·40 (8·18–8·62)	6·14 (5·95–6·32)	1·66 (1·56–1·76)	0·02 (0·01–0·04)	0·05 (0·03–0·07)	0·03 (0·02–0·05)
2009/10	10,637	15·76 (15·46–16·06)	8·22 (8·01–8·44)	5·93 (5·74–6·11)	1·54 (1·45–1·64)	0·02 (0·01–0·03)	0·03 (0·02–0·02)	0·01 (0·01–0·02)
2010/11	11,305	16·56 (16·25–16·86)	8·96 (8·73–9·18)	6·16 (5·98–6·35)	1·37 (1·28–1·46)	0·02 (0·01–0·03	0·02 (0·01–0·04)	0·01 (0·02–0·02)
2011/12	11,947	17·33 (17·02–17·63)	9·35 (9·12–9·58)	6·76 (6·57–6·95)	1·42 (1·36–1·51)	0·02 (0·01–0·04)	0·04 (0·02–0·05)	0·02 (0·01–0·04)
2012/13	13,823	20·17 (19·84–20·51)	10·80 (10·59–11·05)	7·65 (7·44–7·85)	1·63 (1·53–1·72)	0·04 (0·03–0·05)	0·04 (0·03–0·06)	0·02 (0·01–0·03)
2013/14	14,806	22·35 (21·99–22·70)	12·45 (12·19–12·27)	8·22 (8·00–8·36)	1·61 (1·51–1·71)	0·02 (0·01–0·03)	0·03 (0·02–0·04)	0·02 (0·01–0·03)
Cochran Armitage test for trend:		1053·1403 (*P* < 0·0001)	800·999 (*P* < 0·0001)	430·788 (*P* < 0·0001)	0·0260 (*P* = 0·8719)	0·4890 (*P* = 0·4844)	1·4770 (*P* = 0·2242)	1·3500 (*P* = 0·2453)
Feeding difficulties								
2008/09	7581	11·35 (11·10–11·60)	3·94 (3·79–4·09)	4·14 (3·99–4·29)	2·06 (1·95–2·16)	0·77 (0·70–0·83)	0·28 (0·24–0·33)	0·16 (0·13–0·19)
2009/10	8046	11·92 (11·66–12·18)	4·80 (4·63–4·96)	4·12 (3·96–4·27)	1·93 (1·82–2·03	0·68 (0·62–0·74)	0·24 (0·21–0·28)	0·15 (0·12–0·18)
2010/11	8784	12·86 (12·60–13·13)	5·58 (5·40–5·75)	4·27 (4·15–4·43)	1·97 (1·87–2·08)	0·70 (0·64–0·77)	0·22 (0·18–0·25)	0·12 (0·10–0·15)
2011/12	8879	12·88 (12·61–13·14)	5·60 (5·43–5·78)	4·23 (4·08–4·39)	1·95 (1·8–2·05)	0·74 (0·68–0·81)	0·23 (0·19–0·26)	0·13 (0·10–0·15)
2012/13	8706	12·71 (12·44–12·97)	5·75 (5·74–5·93)	4·10 (3·95–4·25)	1·80 (1·70–1·90)	0·70 (0·64–0·77)	0·21 (0·18–0·24)	0·14 (0·11–0·17)
2013/14	8694	13·12 (12·85–13·40)	5·75 (5·56–5·93)	4·29 (4·14–4·45)	1·91 (0·81–2·02)	0·79 (0·73–0·86)	0·25 (0·21–0·29)	0·12 (0·10–0·15)
Cochran Armitage test for trend:		97·70 (*P* < 0·0001)	260·5501 (*P* < 0·0001)	54·0300 (*P* < 0·0001)	6·1920 (*P* = 0·0128)	0·6870 (*P* = 0·4072)	2·7600 (*P* = 0·0966)	3·3200 (*P* = 0·0684)

^a^per 1000 live births

The admission rate for physiological jaundice differed significantly by gender: 44,153 male infants (21·20 per 1000 live births (95% CI 21·03–21·37) were admitted over the period compared to 29,251 female infants (14·77 per 1000 live births (95% CI 14·63–14·92). The infant admission rate for physiological jaundice varied by IMD quintile (Table 4), the lowest in the most deprived quintile (16·97 per 1000 live births, 95% CI 16·73–17·21)). The rate of admission for physiological jaundice differed by ethnicity (Table 4). The lowest rate was for black infants where the rate was 6·97 per 1000 live births (95% CI 6·62–7·33) and the rate of admission was four times higher for infants with an ethnicity code ‘not stated’ (26·14 per 1000 live births, 95% CI 25·41–26·87).

**Table 4:** Number and incidence (per 1000 live births) of infant admissions for potentially preventable conditions by Ethnicity, Gender, and IMD quintile 2008/09–2013/14

	Feeding difficulties		Gastroenteritis		Physiological Jaundice	
	No admissions	Rate (95% CI)	No admissions	Rate (95% CI)	No admissions	Rate (95% CI)
Ethnicity^a^						
White	37,746	12·81 (12·68–12·94)	47,616	16·16 (16·01–16·30)	51,082	17·34 (17·19–17·48)
Asian	5102	12·19 (11·86–12·52)	4570	10·92 (10·60–11·23)	9517	22·74 (22·28–23·19)
Black	1415	6·59 (6·25–6·94)	1783	8·31 (7·92–8·69)	1497	6·97 (6·62–7·33)
Other	3391	11·26 (10·89–11·64)	3365	11·18 (10·80–11·55)	6556	21·78 (21·26–22·30)
Not stated	3036	16·69 (16·10–17·28)	3282	18·04 (17·43–18·65)	4756	26·14 (25·41–26·87)
Gender						
Male	26,183	12·57 (12·42–12·72)	32,761	15·73 (15·56–15·90)	44,153	21·20 (21·03–21·37)
Female	24,502	12·37 (12·22–12·53)	27,854	14·07 (13·90–14·23)	29,251	14·77 (14·63–14·92)
IMD Index^a^						
1	12,708	11·31 (11·11–11·50)	19,122	17·01 (16·77–17·25)	19,077	16·97 (16·73–17·21)
2	10,860	11·89 (11·67–12·11)	13,967	15·29 (15·04–15·55)	16,111	17·64 (17·37–17·91)
3	9899	13·14 (12·82–13·40)	10,903	14·47 (14·20–14·74)	14,084	18·69 (18·39–19·00)
4	8952	13·63 (13·35–13·91)	8953	13·63 (13·35–13·91)	12,367	18·83 (18·50–19·16)
5	7995	12·99 (12·71–13·27)	7254	11·78 (11·51–12·05)	11,245	18·27 (17·93–18·60)

^a^Missing data:

Gastroenteritis: 0.7% IMD index, 0.6% Ethnicity

Physiological Jaundice: 0.9% IMD index, 0.6% Ethnicity

Feeding difficulties: 0.8% IMD index, 0.4% ethnicity

The admission rate for *feeding difficulties* rose from 11·35 (95% CI 11·10–11·60) per 1000 live births in 2008/09 to 13·12 (12·85–13·40) in 2013/14 (*P* < 0.0001). The age specific admission rate for feeding difficulties varied considerably over the period. The largest increase in risk of admission over the period was in the 0–6 day age category where there was a 46% increase in 2013/14 compared with 2008/09 (RR 1·46, 95% CI 1·39–1·54) (*P* < 0.0001). Admissions to hospital for feeding difficulties after one month of age were much less common and the rate consistently decreased with age up to one year (Table 3). The median length of admission for feeding difficulties was 1 day and the majority of infants (91·7%) had an admission of 3 days or under.

There was no significant difference in the rate of admission by gender: the rate for male infants was 12·57 per 1000 live births (95% CI 12·42–12·72) compared to 12·37 per 1000 live births (95% CI 12·22–12·53) for females. There was a small but significant difference in the admission rate for feeding difficulties by IMD quintile with the lowest rate in the most deprived quintile 11·31 per 1000 live births, (95% CI 11·11–11·50). The lowest rate of admission was observed for black infants (6·59 per 1000 live births, 95% CI 6·26–6·94) compared to 16·69 in the ‘not stated’ ethnicity category (95% CI 16·10–17·28).

For *gastroenteritis* the rate of infant admission per 1000 live births rose from 12·14 in 2008/09 (95% CI 11·88–12·40) to 19.86 (95% CI 19·52–20·19) (*P* < 0.0001). The rate of admission for gastroenteritis significantly increased across all age categories but admission was least frequent in infants in the first 28 days. It was greatest in the 9–12 month age category, although infants aged 1–3 months had the largest relative increase in risk of admission (RR 2·04, 95% CI 1·90–2·19) from 2008/09–2013/14 (Table 3). The median length of stay was less than one day and 96.8% infants were discharged within 3 days.

There was a small but significant difference in rate of admission for gastroenteritis by gender; the rate for male infants was 15·73 per 1000 live births (95% CI 15·56–15·90) and 14.07 (95% CI 13·90–14·23) for female infants. The highest rate was noted in the most deprived IMD quintile (17·01 per 1000 live births, 95% CI 16·77–17·25) (Table 4). There was also considerable variation by ethnicity, where the rate of admission per 1000 live births was more than double for infants with ‘not stated’ ethnicity, 18·04 per 1000 live births, (95% CI 17·43–18·65) compared to 8·31 per 1000 live births (95% CI 7·92–8·69).

The number of admissions for the conditions identified as potentially avoidable varied considerably with high numbers of admissions to bigger paediatric hospitals.

## Discussion

The rate of hospital admission in the first year of life for the three conditions identified as potentially preventable increased by 39% relative to an overall increase of 6%. Over the first year the biggest increase in admissions occurred in the first 0–6 days and 85% of the increase in this period was for the identified potentially preventable conditions of jaundice, feeding difficulties and gastroenteritis for which admissions rose from 12·36 to 18·23 per 1000 live births.

This study used a large routinely collected national dataset and a robust method to develop a working definition of ‘potentially avoidable’ infant admissions in the context of postnatal care provision, drawing on the expertise of paediatricians, research data analysts and clinical coders. The potentially avoidable conditions were pre-specified prior to calculation of admission rates. The coding framework used to identify such admissions incorporated inclusion and exclusion criteria to ensure that infants with underlying conditions were excluded from the sample population (for example, infants born with cleft lip and palate, and subsequent feeding difficulties). It is reassuring that the incidence of admissions for physiological jaundice and feeding difficulties over the age of 3 months was very small, suggesting that the selection of codes for these conditions was accurate. Although a systematic review of coding accuracy studies suggested that HES data has improved significantly over time [16], it is unlikely that this would have affected our study findings because the NHS Payment by Results system, a key driver for improving HES data accuracy, had been fully implemented by 2007 [17, 18].

HES is widely accepted as a database for health research and suitable for studies identifying trends in healthcare [19], although there are a number of limitations. The ethnicity variable was not as complete as other data fields with 7% of infant admissions having a ‘missing’ or ‘not known’ code. Previous research has indicated that missing ethnicity data may not be random and instead relates to service pressures, a lack of opportunity for health professionals enquiry or the circumstances of hospital admission [20, 21]. Additionally, the broad denominator ethnicity categories necessary to maintain confidentiality prohibited a thorough assessment of admission rates by ethnicity. It was not possible to explore hospital level admission rates because denominator data were not available at hospital level but we anticipate that variation would be affected by patient and hospital level factors. Finally, we did not have data on smoking status and breastfeeding status.

Use of age specific admission rates for infants under one year showed that the increase in admission over the period 2008 to 2014 only existed within the first 6 months of life, and had increased most in the 0–6 day category. The admission rate for infants from 6 to 12 months remained stable over the period. Our findings are consistent with those of other studies that explore unplanned infant admissions to hospital [6]. It is also consistent with the literature in the finding that the rate of admissions varied by IMD [22]. The overall admission rate to hospital by IMD quintiles supports existing evidence that admission rates are strongly correlated with measures of social deprivation [22]. For admission rates for jaundice and feeding difficulties however the admission rate was highest in the least deprived quintiles and may reflect variation in infant feeding practices with women in the least deprived quintiles more likely to breastfeed. Inability to initiate and establish breastfeeding resulting in an insufficient milk supply is a known risk factor for physiological jaundice [23]. Exclusive initial breastfeeding initially rose from 65% in 2005 to 69% in 2010 when 46% of babies were still exclusively breastfed at one week [24]. While breastfeeding may be a factor influencing the trends seen, it does not provide a sufficient explanation of them. Increases in admission rates for gastroenteritis showed a different pattern from jaundice and feeding difficulties as the increase for this was greatest in infants after the first month and may possibly be related to feeding practices and insufficient support for infant feeding.

The change in infant admission rates we observed over the period was concentrated in those under 7 days of age and for the potentially avoidable conditions, particularly jaundice and feeding difficulties. In England over a similar period of time women and infants have had less routine contact with health professionals as the length of stay in hospital after birth and the median community visits following discharge from birth has reduced [9, 10]. Over the period of this study, the average postnatal length of stay hospital reduced slightly from 1.7 days in 2008/09 to 1.5 days in 2013/14 [25]). Several large surveys of women’s experiences of postnatal care have shown that a large proportion felt that they needed more support, particularly establishing breastfeeding [11, 26–29]. Although temporal association does not prove causation, the increase in admissions may in part prove to be attributed to changes in the postnatal care provision and management of neonates in the community. Other possible causes to the increase observed in this study include an increase in parents being advised by NHS 111 system to take their child straight to hospital, and a decrease in training and experience for doctors to triage neonates in primary care [3]. If the reduction in postnatal care provision does have a part to play in the increase in infant admission rate, the current National Maternity Review in England [11] aimed at transforming maternity services has the opportunity to ensure that women’s needs are being met prior to discharge from hospital. It could also ensure that women are able to have more effective community provision including more frequent home visits where needed and easy access to midwifery advice in order to identify potential infant health problems to improve this situation.

## Conclusion

Our findings show that most of the increase in the rate of admission to hospital for infants up to age one over the period 2008–2014 was in the early neonatal period; and the great majority of this increase is explained by the three conditions, physiological jaundice, feeding difficulties and gastroenteritis, predominantly the former two. Potential missed opportunities within the postnatal care pathway require urgent modification given current NHS capacity and resource issues.

## Additional files

Additional file 1: Flow chart of the process for identifying infant admissions under the age of 1 year unrelated to birth admissions in Hospital Episode Statistics. (DOCX 43 kb)

Additional file 2: Coding framework for potentially avoidable infant admissions. (DOCX 17 kb)

## Abbreviations

GOR: Local Authority District and Government Office Region
HES: Hospital Episode Statistics
IMD: Index of multiple deprivation
LSOA: Lower super output area
ONS: Office for National Statistics
WHO: World Health Organisation
