# Supplementary material for: Hospitalisation after birth of infants: cross sectional analysis of potentially avoidable admissions across England using hospital episode statistics
Source: BMC Pediatr. 2018 Dec 20;18:390. doi: 10.1186/s12887-018-1360-z (PMC6302406; doi:10.1186/s12887-018-1360-z)
Supplement: Supplementary file 1 — Flow chart of the process for identifying infant admissions under the age of 1 year unrelated to birth admissions in Hospital Episode Statistics. (DOCX 43 kb) [file 12887_2018_1360_MOESM1_ESM.docx]

Additional file 1. Flow chart of the process for identifying infant admissions under the age of 1 year unrelated to birth admissions in Hospital Episode Statistics:

Episodes of care involving a patient <12 months of age admitted between 01/04/2008-31/03/2014

n=6,145,402

Excluded

Admission date >01/04/2014

AND/OR

Duplicate cases

AND/OR

Invalid admission/discharge dates:

n=106,739

Excluded

Episodes of care that contained ‘live birth’ as diagnosis code:

n=10,447

Excluded

Episodes of care without admission code ‘elective’ or ‘emergency’:

n=505,610

Excluded

Episodes of care with a code with ‘born in hospital’ or ‘on way to hospital’:

n=4,135,275

Discharge episodes of the spells containing the episodes where the admission date was <01/04/2014

n=1,387,331

Episodes of care without ‘live birth’ as diagnosis code

n=1,494,070

Episodes of care with admission code ‘elective’ or ‘emergency’

n=1,504,517

Episodes of care involving a patient <12 months and without a code related to ‘baby born in hospital’ or ‘on way to hospital’

n=2,010,127
