# Supplementary material for: Hospitalisation after birth of infants: cross sectional analysis of potentially avoidable admissions across England using hospital episode statistics
Source: BMC Pediatr. 2018 Dec 20;18:390. doi: 10.1186/s12887-018-1360-z (PMC6302406; doi:10.1186/s12887-018-1360-z)
Supplement: Supplementary file 2 — Coding framework for potentially avoidable infant admissions. (DOCX 17 kb) [file 12887_2018_1360_MOESM2_ESM.docx]

Additional file 2 Coding framework for potentially avoidable infant admissions

| Condition | Inclusion criteria | Exclusion criteria |
| --- | --- | --- |
| Physiological jaundice | **Primary or secondary ICD-10 diagnosis code :**  P593 Neonatal Jaundice from breastmilk inhibitor  P599 Neonatal Jaundice, unspecified  R17X Unspecified Jaundice  **OPCS4 primary and Secondary code:**  S128 unspecified phototherapy  S129 other phototherapy | **Primary or secondary ICD-10 diagnosis code:**  Q44 Congenital malformations of gallbladder, bile ducts and liver  (Includes atresia of bile ducts, cystic disease of liver, and congenital malformation of bile ducts and more).  E806 Other disorders of bilirubin metabolism (including Dubin-Johnson syndrome and Rotor syndrome)  P070 Extremely low birth weight <999g  P072 Extreme prematurity (<28 completed weeks gestation)  P073 Other preterm infant  P590 Jaundice associated with preterm delivery  **Primary or Secondary OPCS4 code**:  J272 Partial excision of bile duct and anastomosis of bile duct to duodenum  J273 Partial excision of bile duct and anastomosis of bile duct to jejunum  J274 Partial excision of bile duct and end to end anastomosis of bile duct  J278 Other specified excision of bile duct  J279 excision of bile duct |
| Feeding difficulties | **Primary or secondary ICD-10 diagnosis code:**  P922 Slow feeding of newborn  P923 Underfeeding of newborn  P924 Overfeeding of newborn  P925 Neonatal difficulty feeding at breast  P928 Other feeding problems of newborn  P929 Feeding problem of newborn, unspecified  R633 Feeding difficulties and mismanagement (excludes newborn feeding difficulties but is often miscoded) | **Primary or secondary ICD-10 diagnosis code:**  Q35-Q38 Cleft lip and palate malformations  Q38-Q45 Congenital malformation of the digestive system (includes pyloric stenosis, hiatus hernia, and malformation of stomach, atresia and stenosis of duodenum and jejunum and Ileum).  G70-G073 Disease of myoneural junction and muscle (including muscular dystrophy, myotonic dystrophy, congenital myopathy).  Q871 Congenital malformation syndromes predominantly associated with short stature (including Prader-Willi Syndrome)  P070 Extremely low birth weight <999g  P072 Extreme prematurity (<28 completed weeks gestation)  P073 Other preterm infant |
| Gastroenteritis | **Primary or Secondary ICD-10 diagnosis code:**  A080 Rota viral enteritis  A081 Acute gasteropathy due to Norwalk agent  A082 Adenoviral enteritis  A083 Other viral enteritis  A084 Viral intestinal infection, unspecified  A085 Other specified intestinal infections  A090 Other and unspecified gastroenteritis and colitis of infectious origin  A099 Gastroenteritis and colitis of unspecified origin | **Primary and secondary ICD-10 codes:**  K529 Non infective gastroenteritis and colitis, unspecified (diarrhoea, enteritis, ileitis, jejunitis, sigmoiditis)  K528 Other specified non infective gastroenteritis and colitis  Q38-Q45 Congenital malformations of the digestive system (includes pyloric stenosis, hiatus hernia, malformation of stomach, atresia and stenosis of duodenum, jejunum and ileum).  P070 Extremely low birth weight <999g  P072 Extreme prematurity (<28 completed weeks gestation)  P073 Other preterm infant |
